# Early risk stratification of postoperative pneumonia after brain tumor surgery using routine perioperative variables: development and prospective multicenter validation of an interpretable prediction model

**DOI:** 10.3389/fcimb.2026.1863501

**Published:** 2026-08-10

**Authors:** Hongliang Mao, Fengchun Mu, Xinyu Wang, Xin Zhang, Dongfang Meng, Chen Yang, Ming Shan, Jinghai Wan, Ming Yang

**Affiliations:** 1 Department of Neurosurgery, National Cancer Center/National Clinical Research Center for Cancer/Cancer Hospital, Chinese Academy of Medical Sciences and Peking Union Medical College, Beijing, China; 2 The Fourth People’s Hospital of Jinan, Jinan, China; 3 Department of Radiation Oncology, Shandong Cancer Hospital and Institute, Shandong First Medical University and Shandong Academy of Medical Sciences, Jinan, China; 4 Department of Neurosurgery, the First Affiliated Hospital of Anhui Medical University, Hefei, China

**Keywords:** brain tumor surgery, clinical web application, logistic regression, machine learning, nomogram, postoperative pneumonia, SHAP interpretability

## Abstract

**Background:**

Early postoperative pneumonia (POP) is a common and serious complication after brain tumor surgery, but early recognition is difficult because postoperative neurological dysfunction and respiratory symptoms are often non-specific. Existing models are mostly retrospective, not designed for neurosurgical patients, and rarely prospectively validated across centers. We aimed to develop an interpretable model for early POP risk stratification.

**Methods:**

We used routine perioperative data from 1,856 patients undergoing brain tumor surgery at multiple centers in China between 2022 and 2025. Ten machine learning algorithms were compared. From 41 candidate variables, 11 predictors were selected using correlation analysis and LASSO. The final locked model was prospectively tested in one internal temporal cohort and three external cohorts. Performance was assessed by AUC, calibration, and decision curve analysis. Interpretability was evaluated using SHAP, a nomogram, and a web calculator.

**Results:**

Logistic regression showed the best overall performance, with an AUC of 0.897 (95% CI, 0.842–0.952) in the internal cohort and a mean AUC of 0.876 ± 0.044 across the three external cohorts. Key predictors included chronic lung disease (CLD), diabetes mellitus (DM), body mass index (BMI), admission Karnofsky Performance Status (KPS), and preoperative albumin (Alb) and glucose (Glu).

**Conclusion:**

This interpretable 11-variable model enables early POP risk stratification after brain tumor surgery and may support timely preventive intervention in neurosurgical care.

## Introduction

Postoperative pneumonia (POP) is a common and clinically significant complication following brain tumor surgery, leading to prolonged hospitalization, higher mortality, and increased healthcare costs (Russotto et al., 2019). According to the Centers for Disease Control and Prevention (CDC) criteria, POP is diagnosed when new or progressive pulmonary infiltrates appear on imaging, accompanied by fever, respiratory symptoms, or elevated white blood cell or neutrophil counts (Horan et al., 2008). Most cases occur within 3–7 days after surgery, yet timely diagnosis is particularly challenging because reduced consciousness, dysphagia, ventilatory dependence, and postoperative sedation can mask early respiratory deterioration in neurosurgical patients (Wang et al., 2021). Delayed recognition may in turn postpone targeted prevention and escalation of supportive care.

Despite advances in perioperative management, the reported incidence of POP after brain tumor surgery remains high (0.6%–18.3%) (Zhang et al., 2020; Guo et al., 2022; Lan et al., 2025), reflecting persistent challenges in prevention and early detection. Traditional risk scores and regression-based tools have limited accuracy and are typically derived from single-center retrospective cohorts without external validation (Canet et al., 2010; Lan et al., 2023; Xiang et al., 2024b). Thus, an effective and generalizable early POP scoring system is currently lacking. Manual chart review remains the standard for surveillance but is labor-intensive, subjective, and difficult to scale. These limitations underscore the need for automated, reliable, and generalizable methods that can identify high-risk patients before clinical deterioration.

With the rapid development of electronic medical records (EMRs), machine learning (ML) has emerged as a powerful approach for clinical prediction modeling, which offers a promising data-driven approach by capturing nonlinear interactions among perioperative variables and enabling individualized prediction (Koyner et al., 2016; Churpek et al., 2020; Gao et al., 2022). Currently, several ML based models have been developed to predict postoperative complications such as sepsis or pulmonary infection (Jing et al., 2021; Xiang et al., 2024b; Zhao et al., 2024; Chen et al., 2025; Han et al., 2025). However, few studies have developed interpretable ML models specifically for early POP prediction of brain tumor surgery, and even fewer have been externally validated. The multicenter ML model developed by Xu et al. has demonstrated considerable potential in predicting POP, thereby substantiating the feasibility and transferability of data-driven risk stratification strategies (Xu et al., 2025). Nevertheless, translational efforts (or research on practical clinical implementation) remain insufficient.

To address these limitations, we developed and validated an interpretable prediction model for early POP after brain tumor surgery using routinely available perioperative variables. We then evaluated its performance across internal temporal and external geographical prospective cohorts. To support clinical interpretation and implementation, the final model was further presented using SHAP (Lundberg and Lee, 2017), a nomogram, and a web-based calculator.

## Methods

### Study design and participants

This multicenter study was conducted in accordance with the Declaration of Helsinki and approved by the institutional review boards of all participating centers: the Cancer Hospital, Chinese Academy of Medical Sciences and Peking Union Medical College (Ethics ID: 26/050-5775), the First Affiliated Hospital of Anhui Medical University (Ethics ID: 20230224), Shandong Cancer Hospital (Ethics ID: SDTHEC202512005), and Jinan Fourth People’s Hospital (Ethics ID: LL20240079). Written informed consent was waived for the retrospective derivation cohort because only routinely collected de-identified data were analyzed. Written informed consent was obtained for the prospective validation cohorts.

The study comprised one retrospective derivation cohort and four prospective validation cohorts, including one internal temporal cohort and three external cohorts. Consecutive patients who underwent craniotomy for intracranial brain tumor resection at the Cancer Hospital, Chinese Academy of Medical Sciences and Peking Union Medical College between 1 January 2022 and 31 October 2024 were retrospectively identified and used for model development. For temporal validation at the same center, we prospectively enrolled consecutive eligible patients between 1 November 2024 and 30 April 2025 (test P). For external validation, three independent neurosurgical centers prospectively recruited consecutive eligible patients during predefined study periods: the First Affiliated Hospital of Anhui Medical University (1 August 2024–30 April 2025, test A), Shandong Cancer Hospital (1 August 2024–30 April 2025, test S), and Jinan Fourth People’s Hospital (1 August 2024–30 April 2025, test J). These cohorts were used solely to evaluate the final locked model.

Because the prospective cohorts were intended for validation rather than hypothesis testing of an intervention, no formal power-based sample size calculation was performed. Instead, consecutive enrollment within prespecified recruitment windows was adopted at each center. The final study population comprised 1,856 patients, including 242 early POP events. For the final 11-predictor model, the overall events-per-variable ratio was 22 (242/11), exceeding commonly recommended thresholds for multivariable prediction modeling (Riley et al., 2020).

Patients were eligible if they (1) underwent craniotomy for brain tumor resection and (2) had complete data for all candidate predictors. Exclusion criteria included (1) non intracranial tumors, (2) pre-existing pulmonary or systemic infection, (3) recent thoracic surgery or trauma within 30 days, (4) incomplete or inconsistent medical records, and (5) expected survival <7 days or hospice care. During the study period, patients who underwent craniotomy for brain tumor resection at the participating centers and met the eligibility criteria were consecutively screened and included.

**Figure 1:**
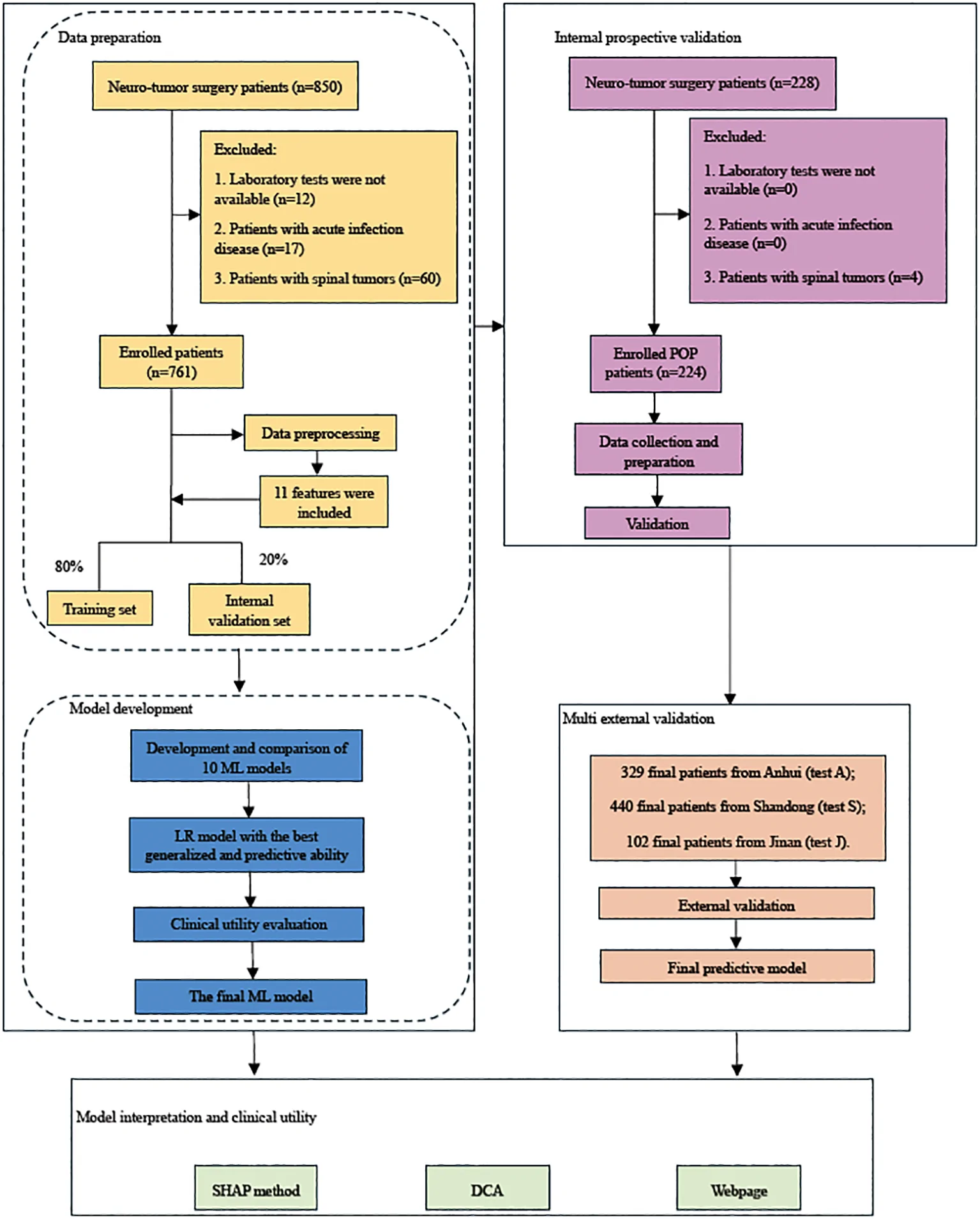
Flow chart of the study design. POP, Postoperative pneumonia; GBM, Gradient boosting machine; SHAP, SHapley Additive explanation.

Early POP was defined *a priori* according to CDC criteria as a new or progressive pulmonary infiltrate on chest radiography or computed tomography within 7 days after surgery, together with compatible clinical manifestations such as fever, purulent sputum, leukocyte abnormality, or worsening oxygenation. To improve inter-center consistency, we used a standardized case report form and a prespecified adjudication checklist. The patient flow diagram (**Figure 1**) summarizes the selection process, including reasons for exclusion at each stage.

### Data collection and optimal feature selection

Candidate predictors were extracted from structured electronic medical records according to their temporal availability, including admission, preoperative, intraoperative, and early postoperative variables up to postoperative day 3. To avoid information leakage, only variables available before clinical or radiological evidence of POP were used; values recorded after POP diagnosis were excluded. These included demographic characteristics, comorbidities, tumor- and surgery-related factors, perioperative management, and routine laboratory indices (**Supplementary 1**, **Supplementary Table 1**).

All candidate predictors were standardized to a z-score distribution (mean = 0, SD = 1) to ensure consistency in the scales between different features.

To address class imbalance, the Synthetic Minority Over-sampling Technique (SMOTE) was applied only to the training data, thereby avoiding data leakage. Multicollinearity was evaluated using Spearman’s correlation analysis. When two variables were highly correlated (|r| > 0.6), the feature less strongly associated with the outcome was removed. LASSO-regularized logistic regression with five-fold cross-validation was then used for further feature selection, and the optimal regularization parameter (λ) was selected according to the minimum cross-validated deviance (Chawla et al., 2002).

### Model development and comparison

The retrospective derivation cohort was randomly divided into a training set (80%) and an internal hold-out test set (20%) using simple random splitting without outcome stratification. The training set was used for feature selection, model fitting, and hyperparameter tuning, whereas the internal hold-out set was reserved for internal performance assessment. Ten candidate algorithms were compared, including logistic regression (LR), Naïve Bayes (NB), support vector machine (SVM), random forest (RF), extremely randomized trees (ET), eXtreme Gradient Boosting (XGBoost), Light Gradient Boosting Machine (LightGBM), AdaBoost, gradient boosting machine (GBM), and multilayer perceptron (MLP). Candidate models were trained in the derivation cohort and evaluated in the internal hold-out set. The leading models were then compared in the prospective internal cohort, and the final locked model was externally validated without refitting or recalibration. Hyperparameters are listed in **Supplementary 1**, **Supplementary Table 3**. After model comparison, the best-performing and most clinically interpretable model was fixed and applied, without refitting or recalibration, to the four prospective cohorts to assess temporal and geographical generalizability.

Model performance was assessed using AUC, accuracy, sensitivity, specificity, PPV, NPV, F1 score, calibration curves, Brier score, and decision curve analysis (Piovani et al., 2023). AUC confidence intervals were estimated using the DeLong method (DeLong et al., 1988), and confidence intervals for threshold-dependent metrics were calculated using bootstrap resampling. Sensitivity analyses were performed without SMOTE, without postoperative variables, and using alternative risk cutoffs.

### Model explanation

SHAP analysis was used to rank feature importance and explain model predictions at both the population and individual levels. For the final LR model, coefficients were additionally translated into a nomogram to provide a more intuitive bedside representation of the linear risk structure.

### Online application deployment

To support clinical use, the final LR model was implemented as a web-based calculator. By entering the 11 routinely available variables, clinicians can obtain an individualized early POP risk estimate. The tool is intended for risk stratification and does not require patient identifiers.

### Statistical analysis

All statistical analyses and ML model development were performed using Python (version 3.10). Continuous variables were expressed as median and interquartile range (IQR) and compared using the Mann–Whitney U test. Categorical variables were expressed as frequencies and percentages and compared using the chi-square test or Fisher’s exact test, as appropriate. Model discrimination was ranked by AUC, and the optimal cutoff value was determined by the Youden index. All tests were two-sided, and P < 0.05 was considered statistically significant.

## Results

### Patient characteristics

The retrospective derivation cohort included 761 patients who underwent brain tumor surgery. Of these, 609 were assigned to the training set and 152 to the internal hold-out set. Early POP occurred in 46 patients (7.6%) in the training set and 25 (16.4%) in the internal hold-out set. This difference reflected stochastic event imbalance after random splitting without outcome stratification, because both subsets were derived from the same retrospective cohort. Four additional prospective cohorts were used for validation: test P (n = 224, POP incidence 16.5%), test A (n = 329, 10.0%), test S (n = 440, 17.7%), and test J (n = 102, 22.5%). Baseline characteristics are summarized in **Supplementary 1**, **Supplementary Table 2**.

### Feature optimization and model development

As shown in **Supplementary 1**, **Supplementary Figure 1**, correlation analysis was first used to remove multicollinear variables, after which LASSO retained 11 predictors with non-zero coefficients for downstream modeling (**Supplementary 1**, **Supplementary Figures 2A–C**). SMOTE was then applied to the training set to address class imbalance. Ten ML algorithms were trained on the same derivation data and compared (**Supplementary 1**, **Supplementary Table 4**).

**Figure 2:**
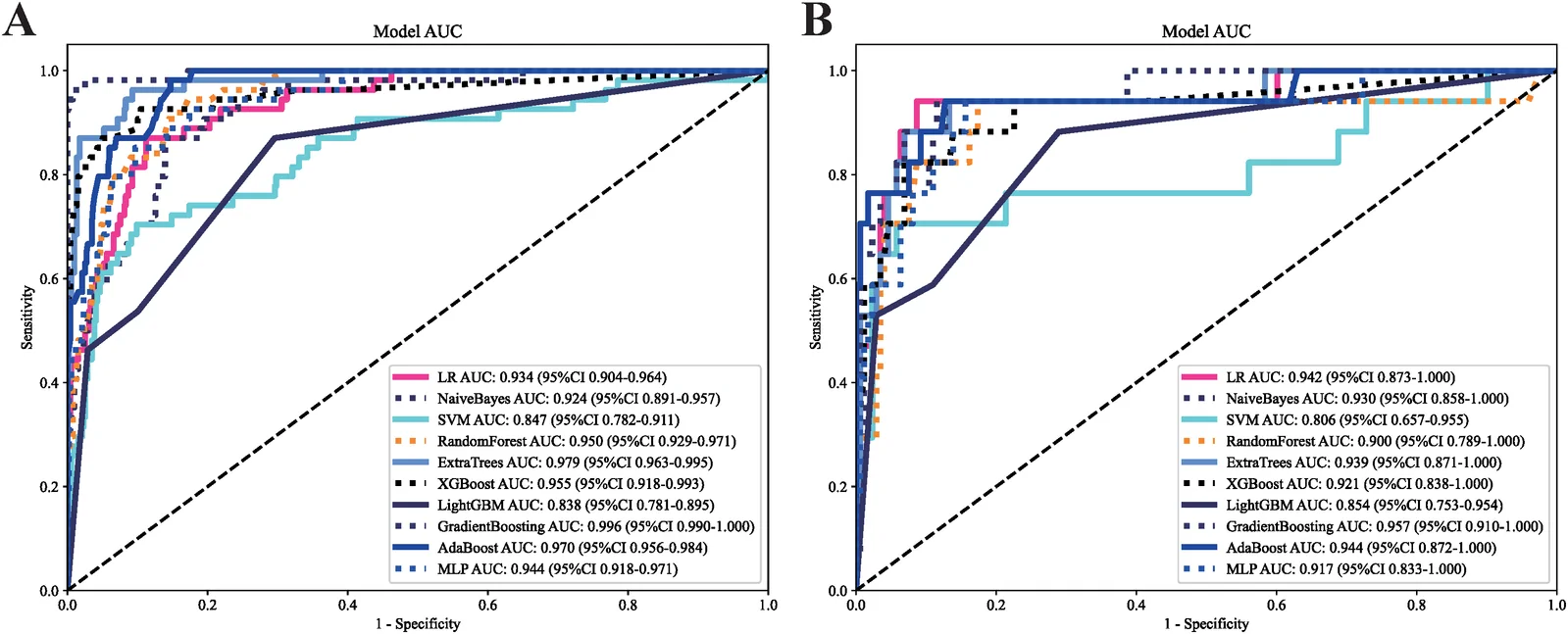
Performance of ML models to predict POP. **(A)** ROC curves and AUC values of 10 models on the training set. **(B)** ROC curves and AUC values of 10 models on the internal validation set. AUC, area under the ROC curve; LR, Logistic regression; SVM, Support vector machine; GBM, Gradient boosting machine; MLP, Multilayer perceptron.

In the training set (**Figure 2A**), GBM achieved the highest apparent performance, with an accuracy of 0.993 and an AUC of 0.996 (95% CI 0.990–1.000). XGBoost and ET also performed well. LR showed stable discrimination, with an AUC of 0.934 (95% CI 0.904–0.964) and an accuracy of 0.901, whereas SVM and LightGBM performed less well.

In the internal validation set (**Figure 2B**), performance declined across all models to varying degrees. GBM, ET, AdaBoost, NB, and LR remained the leading candidates. LR maintained consistent discrimination, with an AUC of 0.942 (95% CI 0.873–1.000) and an accuracy of 0.862. These five models were therefore taken forward for prospective comparison. Sample prediction histograms for those models are shown in **Supplementary 1**, **Supplementary Figure 3**.

### Model comparison and internal prospective validation

**Figure 3:**
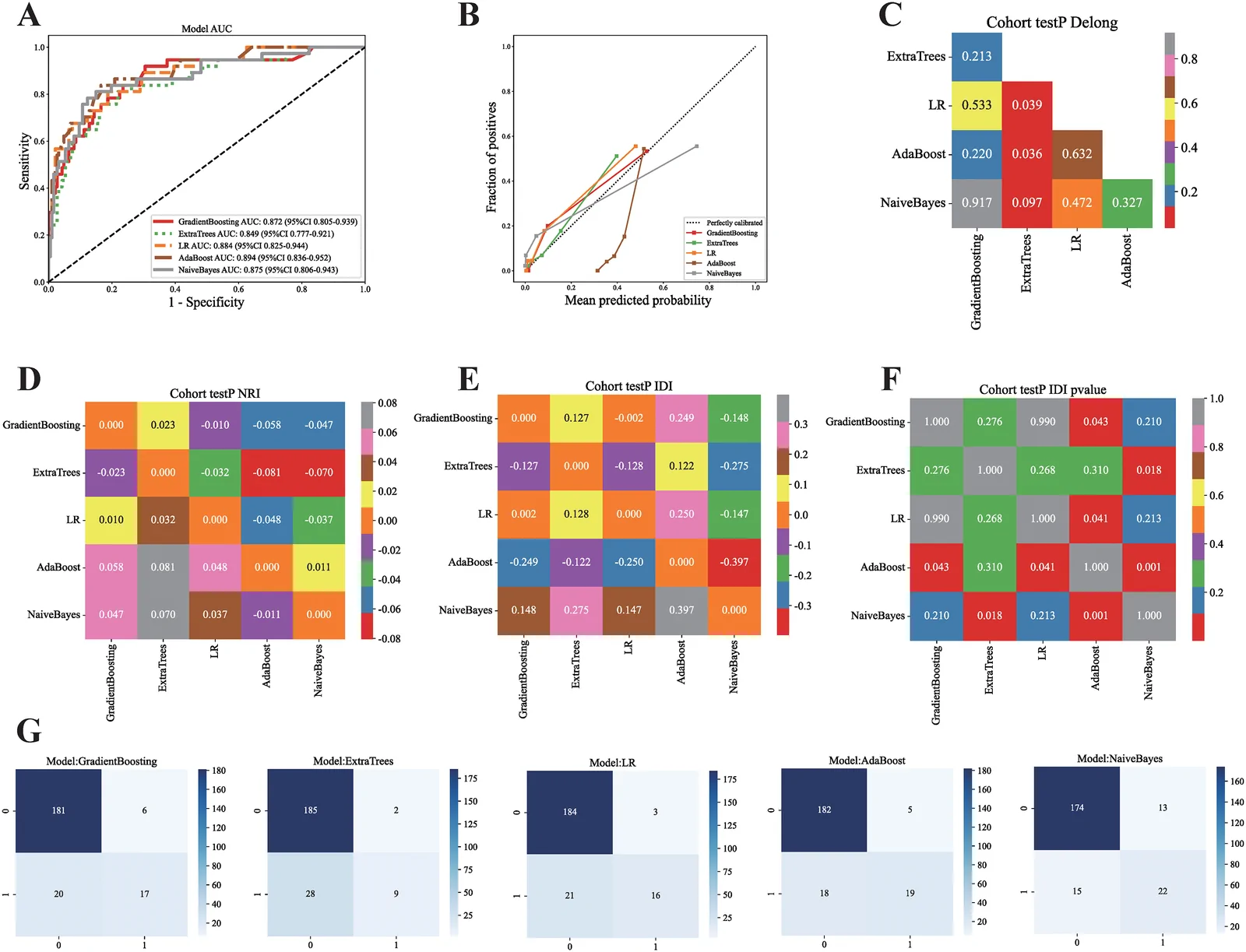
Performance and comparison of the top 5 ML models on the internal prospective validation **(A)** ROC curves and AUC values of the top 5 models on the internal prospective validation. **(B)** Calibration curve analysis of the top 5 models on the internal prospective validation. **(C)** Delong’s test for comparing the AUCs of all possible pairing of the top 5 models. **(D)** NRI comparison for all possible pairing of the top 5 models. **(E)** IDI comparison for all possible pairing of the top 5 models. **(F)** The heatmap of IDI p values. **(G)** Confusion matrix of the top 5 models on the internal prospective validation. NRI, Net reclassification improvement; IDI, Integrated discrimination improvement.

To identify the optimal model for early POP prediction, we compared the five leading models in the internal prospective cohort (test P), focusing on discrimination, calibration, and interpretability. LR showed the best overall performance, with an AUC of 0.897 (95% CI 0.842–0.952), accuracy of 0.853, sensitivity of 0.784, and specificity of 0.866. GBM, NB, ET, and AdaBoost also performed well, but none surpassed LR overall; notably, LR showed better validation performance than GBM in both ROC-AUC and P-R curve assessment. ROC curves are shown in **Figure 3A**, and the P-R curves and detailed metrics are provided in **Supplementary 1**, **Supplementary Figure 3**, **Supplementary 1**, **Supplementary Table 5**, and **Supplementary 1**, **Supplementary Figure 5**, respectively.

Most models showed acceptable calibration, although AdaBoost tended to underestimate event probabilities (**Figure 3B**). DeLong’s test found no statistically significant AUC differences among the five models (**Figure 3C**), and NRI/IDI analyses likewise showed no significant incremental benefit for the alternative models (**Figures 3D–F**). **Figure 3G** presented the confusion matrix distribution for the top 5 models.

**Figure 4:**
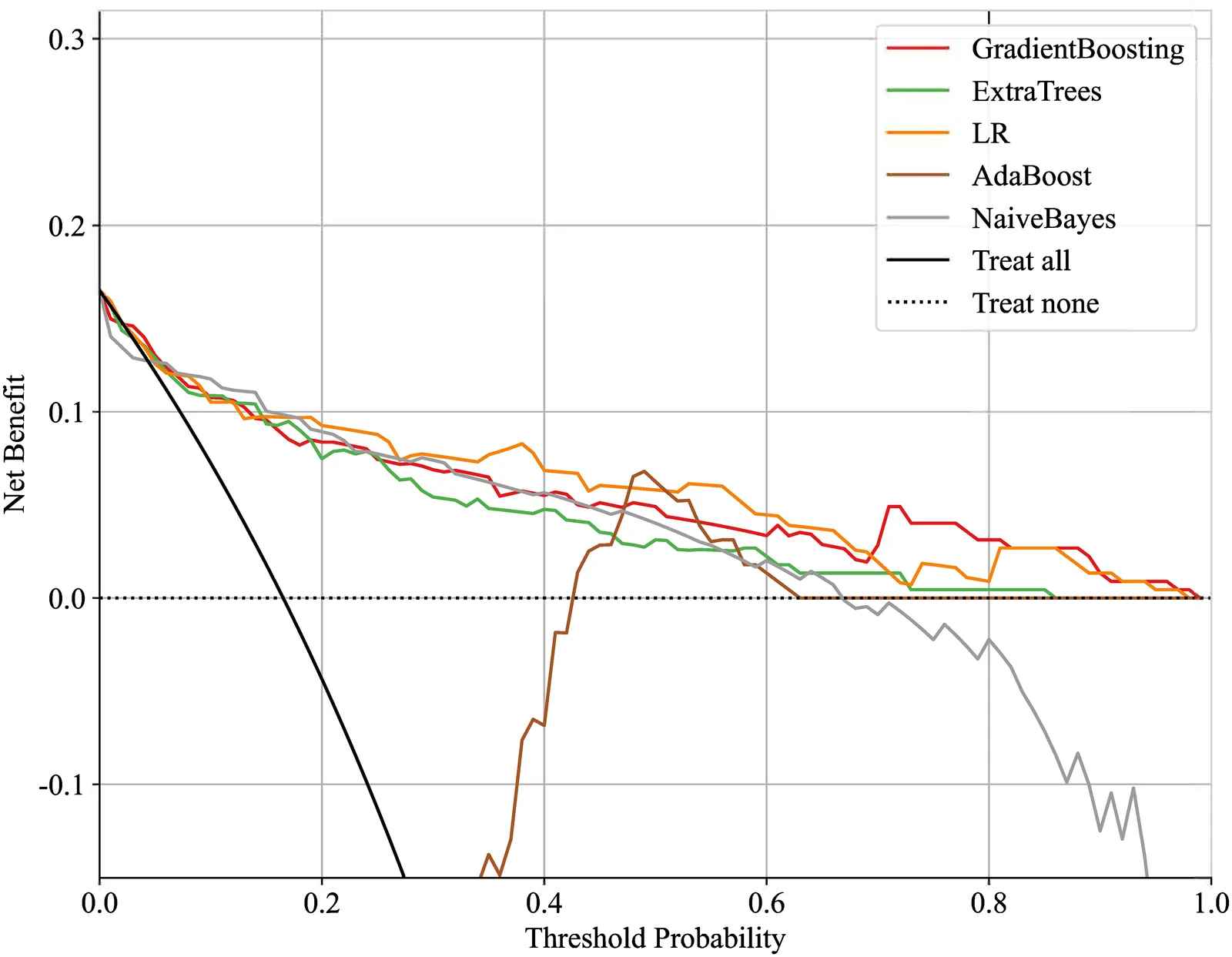
DCA of the final models. The solid black line represents the net benefit of the strategy of treating all patients, and the dashed black line represents the net benefit of the strategy of not treating any patients. DCA, decision curve analysis; LR, Logistic regression.

However, DCA showed that LR provided the highest net clinical benefit across a broad range of threshold probabilities, outperforming both “treat-all” and “treat-none” strategies (**Figure 4**). On this basis, LR was selected as the final model.

### Multi external validation of the final model

**Figure 5:**
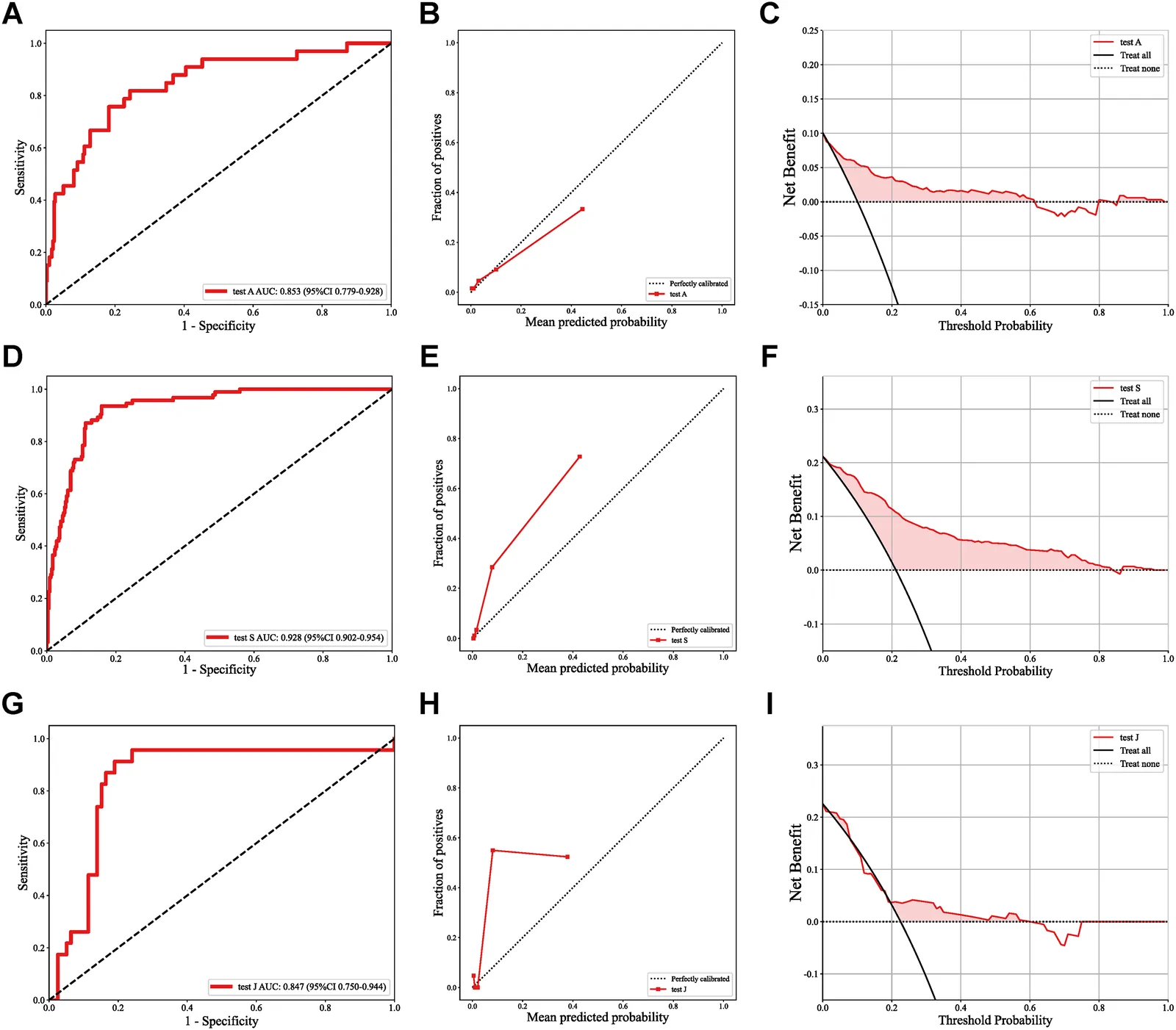
Model performance of LR on test A, test S and test J. **(A, D, G)** ROC curves and AUC values of LR model on test A, test S and test J. **(B, E, H)** Calibration curves of LR model on test A, test S and test J. **(C, F, I)** DCA curves of LR model on test A, test S and test J. DCA, decision curve analysis; LR, Logistic regression.

The locked LR model was then tested in three independent external cohorts. It maintained stable discrimination across centers, with AUCs of 0.853 in test A, 0.928 in test S, and 0.847 in test J (**Figures 5A, D, G**). The pooled mean AUC was 0.876 ± 0.044, comparable to that observed in the internal prospective cohort (ΔAUC = 0.021, p = 0.462). Sensitivity ranged from 0.758 to 0.962 and specificity from 0.823 to 0.866 across centers. Calibration curves showed good agreement between predicted and observed probabilities (**Figures 5B, E, H**), and DCA supported consistent clinical net benefit in all three external cohorts (**Figures 5C, F, I**). These results confirmed the high reproducibility and strong external validity of the LR model across different institutions and patient populations. Additional sensitivity analyses showed that the LR model remained robust without SMOTE, after excluding postoperative variables, and across alternative risk cutoffs (**Supplementary 2**, **Supplementary Tables 1**-**S3**).

### Model explanation

**Figure 6:**
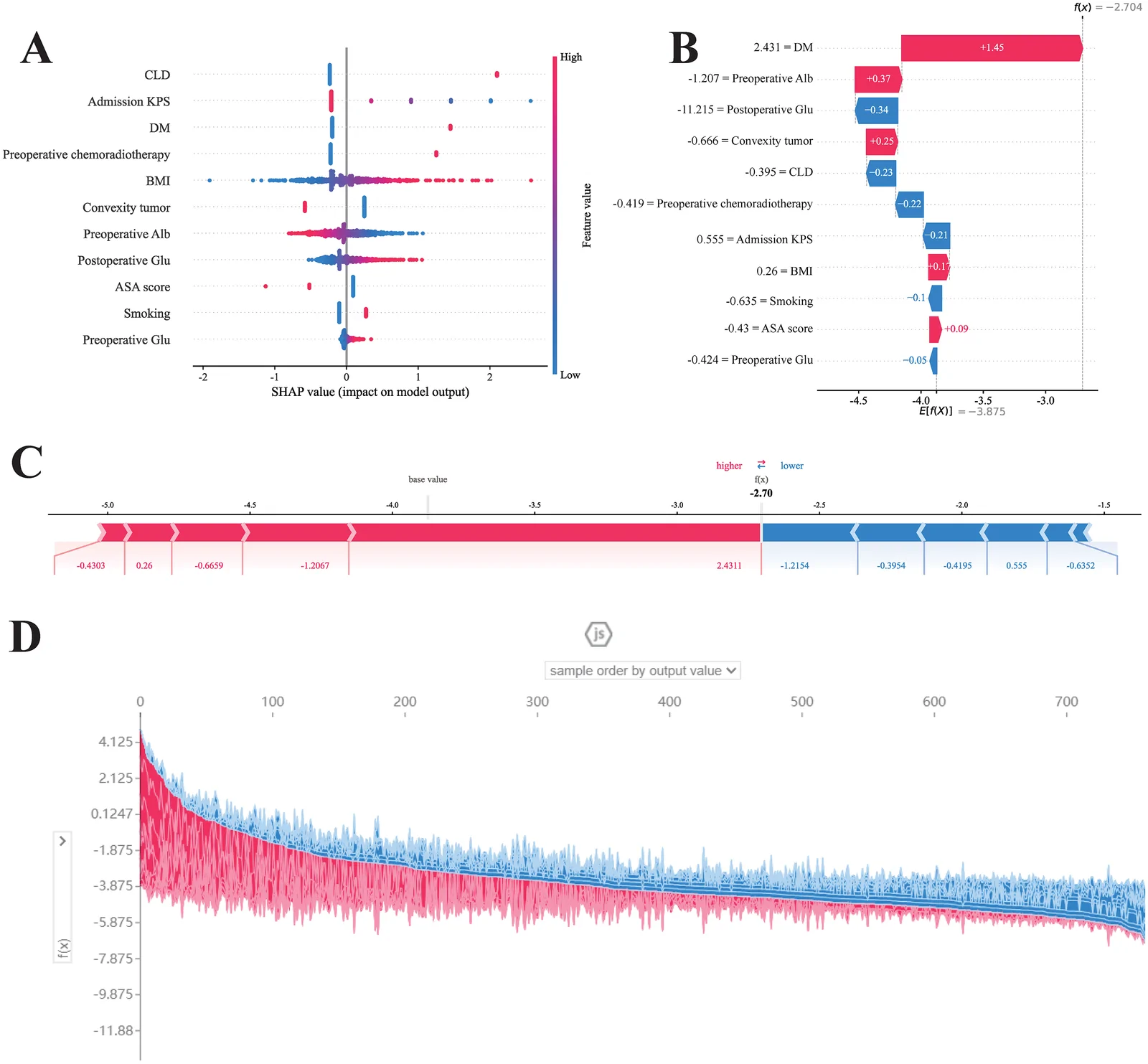
SHAP analysis of the final LR model. **(A)** Global explanation, SHAP summary dot plot. **(B, C)** Local explanation, waterfall plot and evolution of risks contributed by each feature for individual patient at high risk of developing early POP. **(D)** SHAP values for all 609 patients in the training set. CLD, Chronic lung disease; DM, Diabetes mellitus; BMI, Body mass index; KPS, Karnofsky Performance Status scale; ASA, American Society of Anesthesiologists; SHAP, SHapley Additive explanation.

Because LR is inherently interpretable, we further examined the decision logic of the final model using SHAP analysis and a nomogram. The global SHAP summary plot (**Figure 6A**) showed the relative contribution and direction of each predictor to early POP risk. CLD, admission KPS, DM, preoperative chemoradiotherapy, BMI, and convexity tumor location were among the most influential predictors, and dependence plots for all 11 predictors are provided in **Supplementary 1**, **Supplementary Figure 6**, confirming these trends across the full dataset. Local SHAP explanation in a representative high-risk patient showed how individual features cumulatively increased the predicted risk of early POP (**Figures 6B, C**). The distribution of SHAP values across all 609 patients in the training set further demonstrated consistent feature attribution at the population level (**Figure 6D**).

**Figure 7:**
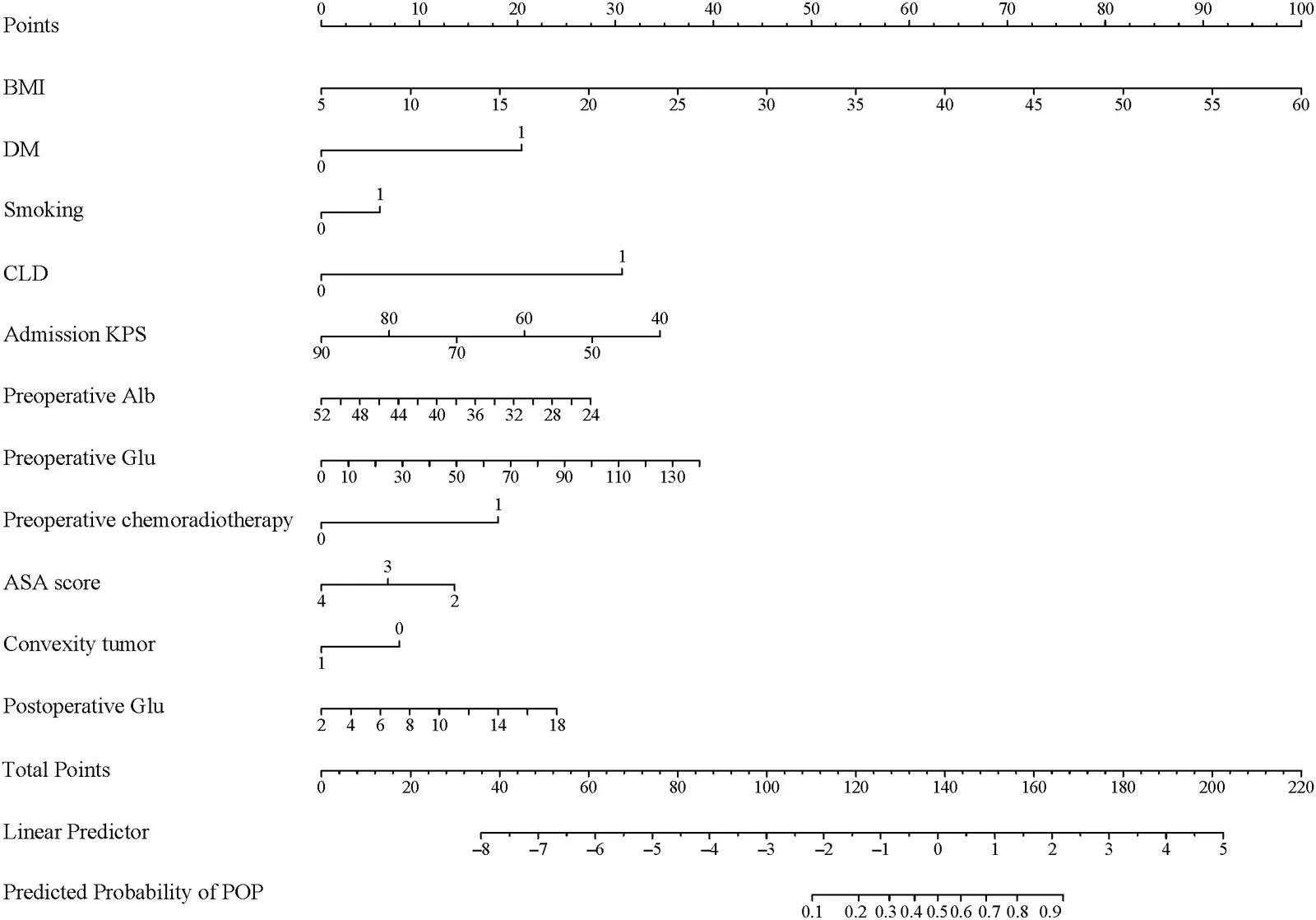
Nomogram for predicting POP after brain tumor surgery based on the LR model. The nomogram integrates 11 clinical predictors—BMI, DM, smoking, CLD, admission KPS, preoperative Alb, preoperative Glu, preoperative chemoradiotherapy, ASA score, convexity tumor, and postoperative Glu—to estimate the individualized probability of POP. Each predictor corresponds to a score on the “Points” scale at the top of the figure. The total points, obtained by summing all variable-specific scores, align with the “Total Points” axis and map to the “Predicted Probability of POP” at the bottom. Higher total points indicate a greater likelihood of postoperative pneumonia. BMI, Body mass index; DM, Diabetes mellitus; CLD, Chronic lung disease; KPS, Karnofsky performance status; ASA, American Society of Anesthesiologists; POP, Postoperative pneumonia.

To facilitate direct clinical application, we visualized the LR model as a nomogram (**Figure 7**). Each predictor corresponds to a specific point value proportional to its regression coefficient, and the total point sum yields an estimated probability of early POP. The bottom scale of the nomogram converts total points into the predicted probability of early POP, providing an intuitive quantitative tool for individualized risk assessment.

### Online application for clinical implementation

**Figure 8:**
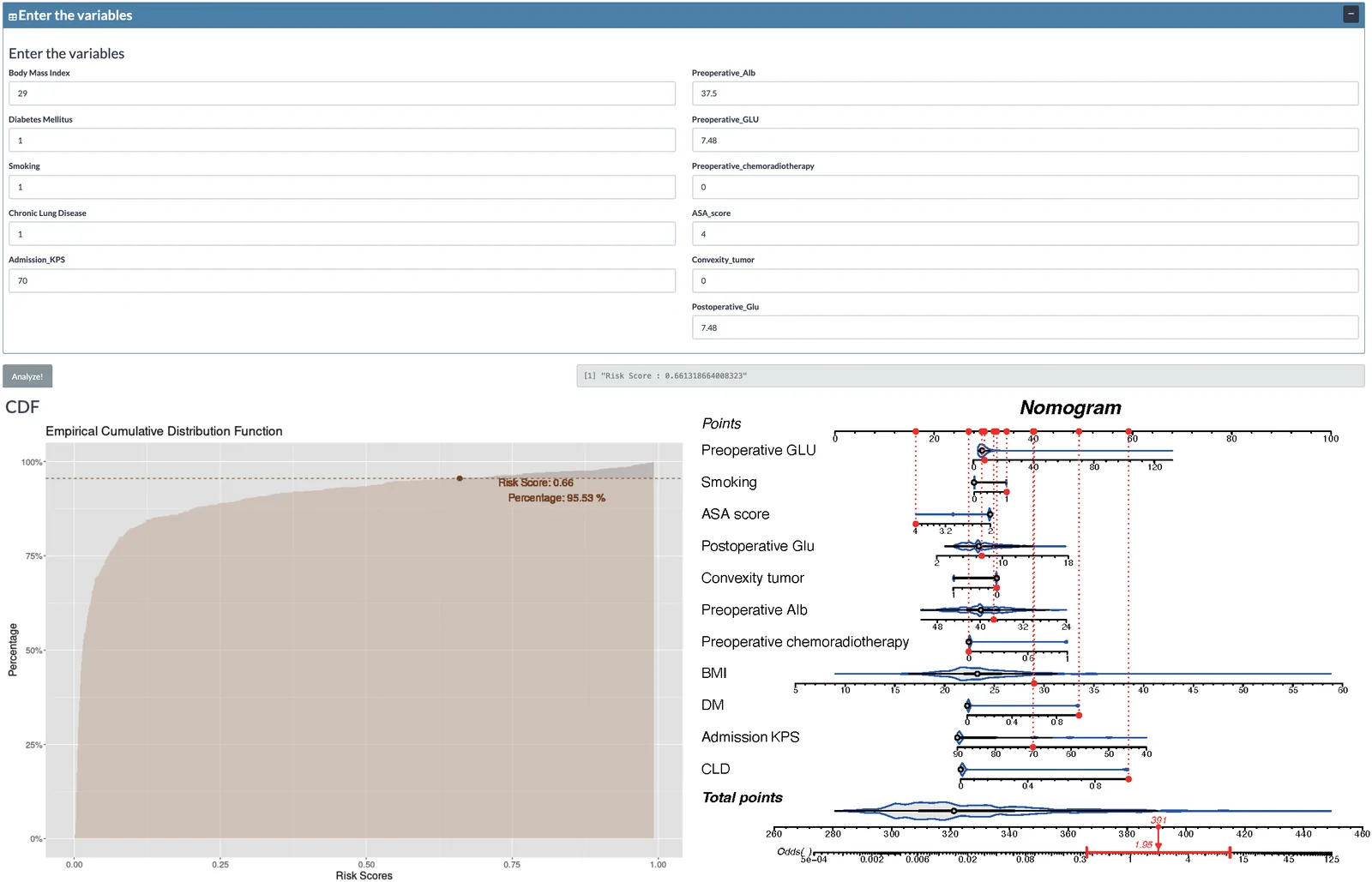
Web-based application for individualized prediction of POP. The final LR model was deployed as an interactive web-based prediction platform to facilitate clinical implementation. Users can input 11 perioperative features to automatically generate a personalized POP risk score. The interface displays the predicted probability along with a cumulative distribution function plot, showing the patient’s relative ranking within the population. Additionally, the system visualizes the nomogram-based computation process and provides the corresponding risk ratio for developing POP. The web application is freely accessible at https://shepherd-ylab.shinyapps.io/POPP, enabling real-time risk prediction and interpretability in clinical settings.

The final LR model was also implemented in an online application, as illustrated in **Figure 8**. By entering 11 routine perioperative variables, clinicians can obtain an individualized early POP risk score, view the patient’s percentile rank within the overall population, and visualize the nomogram-based calculation process. This interface enables rapid bedside risk assessment using routinely available data. The online tool is publicly accessible at https://shepherd-ylab.shinyapps.io/POPP.

## Discussion

In this multicenter study, we developed and validated an interpretable prediction model for early POP after brain tumor surgery using routinely available perioperative variables. The final LR model achieved stable performance, with an AUC of 0.897 in the internal prospective cohort and a mean AUC of 0.876 across three external cohorts. These results suggest that early POP risk can be stratified using variables already available in routine neurosurgical care, without requiring additional invasive testing or specialized data acquisition. The model was further translated into SHAP explanations, a nomogram, and a web-based calculator to support transparent individualized risk assessment.

Our findings extend previous studies on postoperative pulmonary complications. Prior studies have identified CLD (Lee et al., 2019; Zhang et al., 2020; You et al., 2022), DM (Benfield et al., 2007), cardiovascular diseases (Ramalho and Shah, 2021; Wang et al., 2022), age >60 years (Xiang et al., 2022), prolonged operative time (Xiang et al., 2022; Lan et al., 2023) and GCS < 13 (Ding et al., 2017; Mounier et al., 2017) as risk factors for POP. However, their limited feature scope and lack of external validation restricted clinical generalizability (Jing et al., 2021; Xiang et al., 2024b). Beyond neurosurgical cohorts, a previous study proposed a POP model for the general surgical population, defining POP within 30 days postoperatively based on CDC criteria and relying on multi-source EHR extraction (Xiang et al., 2024a), but these models were not specifically designed for brain tumor surgery or for early POP within the first postoperative week. In contrast, our study focused on a clinically actionable early postoperative window and validated the final model across one internal temporal cohort and three external centers.

Although several complex algorithms showed good apparent performance during model development, we selected LR as the final model because it provided the best balance between validation performance, interpretability, calibration, and clinical usability. Apparent training performance alone may overestimate generalizability, particularly for high-capacity algorithms in structured clinical datasets (Arshad et al., 2023; Tan et al., 2025). In addition, excessive model complexity and redundant predictors may reduce the consistency and transportability of individualized clinical risk prediction across heterogeneous settings (Li et al., 2020). In the internal prospective cohort, LR showed favorable validation performance and could be directly translated into a nomogram and web calculator. This transparency is important for bedside use, because clinicians can understand how each predictor contributes to the estimated risk rather than relying on an opaque model output.

The retained predictors are clinically plausible and reflect pulmonary reserve, metabolic vulnerability, nutritional status, and functional impairment. CLD directly increases susceptibility to postoperative respiratory infection, while DM and perioperative hyperglycemia may impair immune response and increase infection risk (Benfield et al., 2007; Lan et al., 2023). Low albumin reflects poor nutritional and inflammatory reserve, and reduced KPS indicates frailty, impaired airway protection, and limited postoperative mobilization (Lim et al., 2012; Li et al., 2015). SHAP analysis confirmed that these predictors contributed consistently to individual- and population-level risk estimates, allowing clinicians to trace the contribution of each variable to an individual prediction and mitigating the ‘black-box’ concern that often hinders ML adoption in healthcare (Azodi et al., 2020). These insights not only validate the biological plausibility of our predictors but also translate directly into actionable targets for perioperative optimization.

Clinically, this model is intended as an early warning and triage tool rather than a standalone diagnostic test. Given the relatively low incidence of early POP, the model showed consistently high NPV but comparatively modest PPV, a pattern commonly observed in prediction models for infrequent clinical events. This suggests that the model may be particularly useful for identifying low-risk patients and supporting early triage, whereas positive high-risk predictions should prompt closer monitoring and preventive assessment rather than be interpreted as definitive POP diagnosis. A high-risk prediction should not automatically trigger empirical antibiotics. Instead, it may prompt more intensive perioperative surveillance and preventive care, including enhanced pulmonary hygiene, aspiration precautions, early mobilization, secretion management, closer monitoring of oxygen saturation and temperature, stricter glycemic control, nutritional assessment, and earlier chest imaging or infection work-up when respiratory symptoms, hypoxemia, fever, or sputum changes emerge. For patients with poor functional status or high aspiration risk, the model may also support earlier swallowing assessment, respiratory physiotherapy, and multidisciplinary review. Conversely, patients predicted to be at low risk may continue routine postoperative surveillance and avoid unnecessary escalation of monitoring or interventions.

Existing scores such as the Canet/ARISCAT score were developed for broader postoperative pulmonary complications in general surgical populations (Canet et al., 2010), and a more recent postoperative pneumonia model also focused on non-neurosurgical populations (Russotto et al., 2019). These tools provide useful clinical context, but they were not specifically designed for early CDC-defined POP after brain tumor surgery, where neurological impairment, airway protection, postoperative consciousness changes, and neurosurgical care pathways are particularly relevant. In addition, several components required for direct score reconstruction were not uniformly available across all participating centers. Therefore, direct head-to-head comparison was not performed. We also did not calculate an approximate or partial score, because incomplete reconstruction could compromise the validity of the comparison and introduce bias. Future prospective studies should collect all required components to enable formal head-to-head comparison with established pulmonary-risk scores.

Several limitations should be acknowledged. First, although the model was externally validated, all participating centers were Chinese tertiary hospitals; therefore, further validation is needed in non-Chinese populations, lower-volume hospitals, and healthcare systems with different perioperative care pathways. Second, the derivation cohort was retrospective, which may introduce selection bias despite subsequent prospective validation. Third, POP diagnosis was based on routine clinical assessment supported by imaging and laboratory evidence. Although CDC-based criteria, standardized case report forms, and prespecified adjudication checklists were used, inter-center differences in imaging frequency, radiographic interpretation, microbiological testing, and diagnostic thresholds may have introduced outcome-label heterogeneity. Fourth, several intraoperative and postoperative process-of-care variables were unavailable or not uniformly recorded across centers, including ventilation parameters, anesthetic management, extubation strategy, aspiration events, secretion management, postoperative swallowing function, rehabilitation intensity, and microbiological data. Some postoperative process variables may also occur close to POP onset, raising temporal ambiguity and potential information leakage if incorporated retrospectively. These variables should be prospectively collected and evaluated in future model updating studies. Finally, because direct comparison with established scores such as Canet/ARISCAT was not feasible in the current dataset, future studies should evaluate whether this neurosurgery-specific model provides incremental value over general pulmonary-risk scores.

## Conclusion

We developed and prospectively validated an interpretable LR model for early POP prediction after brain tumor surgery. Using 11 routinely available perioperative variables, the model achieved good discrimination, acceptable calibration, and stable performance across internal and external cohorts. Combined with SHAP interpretation, a nomogram, and a web-based calculator, it provides transparent individualized risk assessment and may support earlier preventive intervention and more efficient perioperative management in neurosurgical care.

## Data Availability

The original contributions presented in the study are included in the article/**Supplementary Material**, further inquiries can be directed to the corresponding author/s.
