# Supplementary material for: Early risk stratification of postoperative pneumonia after brain tumor surgery using routine perioperative variables: development and prospective multicenter validation of an interpretable prediction model
Source: Front Cell Infect Microbiol. 2026 Aug 10;16:1863501. doi: 10.3389/fcimb.2026.1863501 (PMC13500338; doi:10.3389/fcimb.2026.1863501)
Supplement: Supplementary file 1 [file SupplementaryFile1.docx]

**Supplementary materials 1**

1. Supplementary Table S1: Clinical features of combined retrospective cohort.

2. Supplementary Table S2: Comparison of demographic and clinical features among the training, internal validation, the prospective and external validation cohorts.

3. Supplementary Table S3. Hyperparameters of ten candidate ML models.

4. Supplementary Table S4. Performance of the ML models for early POP prediction in the training and external test cohorts.

5. Supplementary Table S5. Performance of the top 5 ML models for early POP prediction in the prospective external validation dataset.

6. Supplementary Fig. S1 Heatmap of Spearman’s correlation analyses among variables.

7. Supplementary Fig. S2 Feature selection using the LASSO regression model.

8. Supplementary Fig. S3 Distribution of predicted probability scores for top 5 ML models in distinguishing POP from non-POP samples.

9. Supplementary Fig. S4 Precision-recall curves of top 5 ML models in distinguishing POP from non-POP samples in the prospective validation dataset.

10. Supplementary Fig. S5 Distribution of predicted probability scores for top 5 ML models in distinguishing POP from non-POP samples in the prospective external dataset.

11. Supplementary Fig. S6 SHAP dependence plot for the 11 features.

**Supplementary Table S1: Clinical features of the training cohort.**

| **Features** | **Non-pneumonia (N=690)** | **Pneumonia (N=71)** | **p-value** |
| --- | --- | --- | --- |
| Age, year (median, IQR) | 56.0 [45.0, 63.8] | 58.0 [50.5, 67.5] | 0.121 |
| BMI, kg/m² (median, IQR) | 23.1 [22.0, 25.5] | 25.0 [23.0, 27.9] | **< 0.05** |
| Admission GCS, (median, IQR) | 15.0 [15.0, 15.0] | 14.0 [12.0, 15.0] | **< 0.05** |
| Admission KPS, (median, IQR) | 90.0 [90.0, 90.0] | 70.0 [60.0, 85.0] | **< 0.05** |
| Preoperative Alb, g/L (median, IQR) | 40.0 [36.9, 42.6] | 35.0 [32.0, 40.3] | **< 0.05** |
| Preoperative Hgb, g/L (median, IQR) | 134.0 [123.0, 144.0] | 137.0 [125.0, 144.5] | 0.145 |
| Preoperative Glu, mmol/L (median, IQR) | 5.8 [5.1, 7.4] | 5.6 [4.9, 7.4] | 0.672 |
| Preoperative length of hospitalization (median, IQR) | 4.0 [2.3, 6.0] | 4.0 [3.0, 6.0] | 0.518 |
| Preoperative neutrophils, x 10^9^/L (median, IQR) | 4.0 [2.5, 5.8] | 3.4 [2.4, 5.7] | **< 0.05** |
| Preoperative lymphocytes, x 10^9^/L (median, IQR) | 1.8 [1.3, 2.4] | 1.7 [1.2, 2.2] | 0.314 |
| Preoperative monocytes, x 10^9^/L (median, IQR) | 0.44 [0.3, 0.7] | 0.43 [0.3, 0.7] | 0.452 |
| Preoperative SIRI (median, IQR) | 1.0 [0.5, 1.9] | 1.0 [0.6, 1.9] | 0.374 |
| EBL, ml (median, IQR) | 150.0 [50.0, 300.0] | 150.0 [50.0, 300.0] | 0.112 |
| Operating time, min (median, IQR) | 300.0 [240.0, 360.0] | 300.0 [240.0, 420.0] | 0.343 |
| Postoperative Alb, g/L (mean ± SD) | 34.8 [32.0, 37.1] | 33.7 [31.3, 37.1] | 0.129 |
| Postoperative Glu, mmol/L (median, IQR) | 7.0 [5.9, 8.8] | 7.4 [6.1, 9.1] | 0.055 |
| Postoperative neutrophils, x 10^9^/L (median, IQR) | 8.8 [6.3, 11.7] | 8.8 [6.8, 11.6] | 0.425 |
| Postoperative lymphocytes, x 10^9^/L (median, IQR) | 1.3 [0.8, 2.1] | 1.5 [0.9, 2.1] | 0.089 |
| Postoperative monocytes, x 10^9^/L (median, IQR) | 0.6 [0.4, 0.8] | 0.6 [0.4, 0.8] | 0.328 |
| Postoperative SIRI (median, IQR) | 3.4 [1.7, 6.4] | 3.4 [1.6, 6.3] | 0.257 |
| Duration of EVD, days, (median, IQR) | 0.0 [0.0, 0.0] | 0.0 [0.0, 0.0] | 0.060 |
| Duration SCD placement, days, (median, IQR) | 3.0 [2.0, 3.0] | 3.0 [0.0, 3.0] | 0.300 |
| Gender, n (%) |  |  | 0.06 |
| Female | 443 (64.2%) | 37 (52.1%) |  |
| Male | 247 (35.8%) | 34 (47.9%) |  |
| Preoperative CLD, n (%) |  |  | **< 0.05** |
| No | 630 (91.3%) | 20 (28.2%) |  |
| Yes | 60 (8.7%) | 51 (71.8%) |  |
| Preoperative CVD, n (%) |  |  | **< 0.05** |
| NO | 598 (86.7%) | 51 (71.8%) |  |
| Yes | 92 (13.3%) | 20 (28.2%) |  |
| Preoperative DM, n (%) |  |  | **< 0.05** |
| No | 619 (89.7%) | 29 (40.8%) |  |
| Yes | 71 (10.3%) | 42 (59.2%) |  |
| Smoking history, n (%) |  |  | > 0.999 |
| No | 509 (73.8%) | 52 (73.2%) |  |
| Yes | 181 (26.2%) | 19 (26.8%) |  |
| Drinking history, n (%) |  |  | 0.258 |
| No | 602 (87.2%) | 58 (81.7%) |  |
| Yes | 88 (12.8%) | 13 (18.3%) |  |
| Preoperative chemoradiotherapy, n (%) |  |  | **< 0.05** |
| No | 612 (88.7%) | 35 (49.3%) |  |
| Yes | 78 (11.3%) | 36 (50.7%) |  |
| ASA classification, n (%) |  |  | 0.710^#^ |
| I | 0 | 0 |  |
| II | 575 (83.3%) | 59 (83.1%) |  |
| III | 104 (15.1%) | 12 (16.9%) |  |
| IV | 11 (1.6%) | 0 |  |
| Endoscopic surgery, n (%) |  |  | 0.606^#^ |
| No | 583 (84.3%) | 62 (87.3%) |  |
| Yes | 107 (15.7%) | 9 (12.7%) |  |
| Skull base tumor, n (%) |  |  | 0.764 |
| No | 518 (75.1%) | 55 (77.5%) |  |
| Yes | 172 (24.9%) | 16 (22.5) |  |
| Intracranial-extracranial communicating tumor, n (%) |  |  | 0.812^#^ |
| No | 637 (89.4%) | 67 (94.4%) |  |
| Yes | 53 (10.6%) | 4 (5.6%) |  |
| Craniotomy, n (%) |  |  | 0.718 |
| No | 114 (16.5%) | 10 (14.1%) |  |
| Yes | 576 (83.5%) | 61 (85.9%) |  |
| Cerebral convexity tumor, n (%) |  |  | > 0.929 |
| No | 477 (69.1%) | 50 (70.4%) |  |
| Yes | 213 (30.9%) | 21 (29.6%) |  |
| Supratentorial tumor, n (%) |  |  | 0.579 |
| No | 379 (54.9%) | 36 (50.7%) |  |
| Yes | 311 (45.1%) | 35 (49.3%) |  |
| Infratentorial tumor, n (%) |  |  | 0.853 |
| No | 575 (83.3%) | 58 (81.7%) |  |
| Yes | 115 (16.7%) | 13 (18.3%) |  |
| Pathology classification, n (%) |  |  | 0.240 |
| Brain metastases | 181 (26.2%) | 21 (29.6%) |  |
| Primary malignancy | 179 (25.9%) | 13 (18.3%) |  |
| Primary benign | 330 (47.9%) | 34 (47.9%) |  |
| Postoperative EVD, n (%) |  |  | 0.093^#^ |
| No | 681 (98.7%) | 68 (95.8%) |  |
| Yes | 9 (1.3%) | 3 (4.2%) |  |
| SCD placement, n (%) |  |  | 0.698 |
| No | 165 (23.9%) | 19 (26.8%) |  |
| Yes | 525 (76.1%) | 52 (73.2%) |  |
| Reoperation, n (%) |  |  | > 0.999^#^ |
| No | 638 (92.5%) | 66 (93.0%) |  |
| Yes | 52 (7.5%) | 5 (7.0%) |  |

Continuous values were presented as median [interquartile range, IQR]. Categorical values were presented as number (percentage).

\# Calculate statistics using Fisher’ exact test.

BMI, body mass index; GCS, glasgow coma scale; KPS, karnofsky performance status; Alb, serum albumin; Hgb, hemoglobin; Glu, serum glucose; CRP, C-reactive protein; MV, mechanical ventilation; EVD, extraventricular drainage; SCD, subcutaneous drainage; CLD, chronic lung disease; CVD, cardiovascular disease; DM, diabetes mellitus; SIRI, systemic inflammatory response index; ASA, American Society of Anesthesiologists.

**Supplementary Table S2: Comparison of demographic and clinical features among the training, internal validation and external test cohorts.**

| **Features** | **Training (N=609)** | **Internal validation (N=152)** | **External test P (N=224)** | **External test A (N=329)** | **External test S (N=440)** | **External test J (N=102)** |
| --- | --- | --- | --- | --- | --- | --- |
| Age, year (median, IQR) | 56.0 [46.0, 64.0] | 55.0 [45.8, 64.0] | 56.0 [47.8, 62.3] | 50.0 [44.0, 61.0] | 55.0 [46.0, 62.0] | 59.0 [48.0, 66.8] |
| BMI, kg/m² (median, IQR) | 23.1 [22.0, 25.5] | 24.0 [22.7, 26.6] | 23.5 [21.2, 26.5] | 23.4 [21.3, 26.0] | 23.4 [22.0, 26.4] | 22.0 [21.8, 24.2] |
| Admission GCS, (median, IQR) | 15.0 [15.0, 15.0] | 15.0 [14.0, 15.0] | 15.0 [15.0, 15.0] | 15.0 [15.0, 15.0] | 15.0 [15.0, 15.0] | 15.0 [15.0, 15.0] |
| Admission KPS, (median, IQR) | 90.0 [90.0, 90.0] | 90.0 [70.0, 90.0] | 90.0 [80.0, 90.0] | 90.0 [80.0, 90.0] | 90.0 [80.0, 90.0] | 90.0 [80.0, 90.0] |
| Preoperative Alb, g/L (median, IQR) | 39.9 [36.2, 41.7] | 39.5 [36.2, 41.7] | 38.8 [35.4, 41.0] | 39.5 [36.1, 42.2] | 40.8 [35.4, 41.0] | 41.3 [39.7, 43.4] |
| Preoperative Hgb, g/L (median, IQR) | 135.0 [124.0, 144.0] | 132.0 [120.8, 144.2] | 125.0 [113.0, 134.0] | 131.0 [120.0, 143.0] | 133.5 [122.0, 144.0] | 132.0 [123.0, 142.0] |
| Preoperative Glu, mmol/L (median, IQR) | 5.8 [5.1, 7.5] | 5.6 [5.1, 6.9] | 5.7 [5.2, 6.5] | 5.7 [5.1, 7.1] | 5.9 [5.2, 7.5] | 5.7 [4.9, 6.7] |
| Preoperative length of hospitalization (median, IQR) | 4.0 [3.0, 6.0] | 4.0 [2.0, 6.0] | 4.0 [2.0, 6.0] | 4.0 [2.0, 6.0] | 3.0 [3.0, 3.0] | 3.0 [2.0, 4.0] |
| Preoperative neutrophils, x 10^9^/L (median, IQR) | 4.0 [2.4, 5.8] | 3.8 [2.6, 6.0] | 4.0 [2.5, 6.1] | 4.0 [2.5, 5.8] | 3.5 [2.3, 5.4] | 4.0 [2.3, 5.0] |
| Preoperative lymphocytes, x 10^9^/L (median, IQR) | 1.8 [1.3, 2.3] | 1.8 [1.2, 2.4] | 1.8 [1.3, 2.4] | 1.8 [1.2, 2.3] | 1.9 [1.4, 2.4] | 2.1 [1.5, 2.4] |
| Preoperative monocytes, x 10^9^/L (median, IQR) | 0.4 [0.3, 0.7] | 0.4 [0.3, 0.7] | 0.4 [0.3, 0.7] | 0.4 [0.3, 0.7] | 0.4 [0.3, 0.6] | 0.4 [0.3, 0.6] |
| Preoperative SIRI (median, IQR) | 1.0 [0.5, 1.9] | 1.1 [0.5, 1.9] | 1.0 [0.5, 1.9] | 1.0 [0.5, 1.9] | 0.5 [0.4, 0.7] | 0.5 [0.3, 0.7] |
| EBL, ml (median, IQR) | 150.0 [50.0, 300.0] | 100.0 [50.0, 200.0] | 100.0 [50.0, 200.0] | 300.0 [150.0, 480.0] | 360.0 [280.0, 420.0] | 360.0 [280.0, 445.0] |
| Operating time, min (median, IQR) | 300.0 [240.0, 360.0] | 300.0 [240.0, 360.0] | 240.0 [180.0, 360.0] | 300.0 [240.0, 380.0] | 300.0 [240.0, 350.0] | 300.0 [240.0, 370.0] |
| Postoperative Alb, g/L (median, IQR) | 35.0 [32.3, 37.1] | 33.8 [30.5, 36.3] | 34.0 [31.1, 35.9] | 34.3 [31.9, 37.0] | 35.0 [32.4, 37.3] | 35.1 [31.7, 37.7] |
| Postoperative Glu, mmol/L (median, IQR) | 7.0 [5.9, 8.9] | 7.9 [6.0, 8.9] | 7.9 [7.0, 9.3] | 7.5 [6.1, 9.0] | 7.1 [5.9, 9.4] | 7.0 [5.9, 9.2] |
| Postoperative neutrophils, x 10^9^/L (median, IQR) | 8.8 [6.3, 11.7] | 9.0 [6.6, 11.7] | 9.8 [6.8, 12.3] | 8.4 [6.3, 11.3] | 8.7 [6.3, 11.5] | 8.1 [5.2, 10.4] |
| Postoperative lymphocytes, x 10^9^/L (median, IQR) | 1.3 [0.8, 2.1] | 1.4 [0.9, 2.2] | 1.4 [0.8, 2.1] | 1.3 [0.8, 2.1] | 1.4 [0.9, 2.2] | 1.5 [0.8, 2.0] |
| Postoperative monocytes, x 10^9^/L (median, IQR) | 0.6 [0.4, 0.8] | 0.6 [0.4, 0.8] | 0.6 [0.4, 0.8] | 0.5 [0.4, 0.7] | 0.8 [0.5, 1.4] | 0.9 [0.5, 1.8] |
| Postoperative SIRI (median, IQR) | 3.4 [1.7, 6.3] | 3.2 [1.6, 6.5] | 3.7 [1.9, 7.0] | 3.3 [1.7, 6.0] | 3.3 [1.7, 6.5] | 2.9 [1.7, 5.0] |
| Duration of EVD, days, (median, IQR) | 0.0 [0.0, 0.0] | 0.0 [0.0, 0.0] | 0.0 [0.0, 0.0] | 0.0 [0.0, 0.0] | 0.0 [0.0, 0.0] | 0.0 [0.0, 0.0] |
| Duration SCD placement, days, (median, IQR) | 3.0 [0.0, 3.0] | 3.0 [2.0, 3.0] | 3.0 [0.0, 3.0] | 3.0 [1.0, 3.0] | 3.0 [3.0, 3.0] | 3.0 [2.0, 3.0] |
| POP, n (%) |  |  |  |  |  |  |
| No | 563 (92.4%) | 127 (83.6%) | 187 (83.5%) | 296 (90.0%) | 362 (82.3%) | 79 (77.5%) |
| Yes | 46 (7.6%) | 25 (16.4%) | 37 (16.5%) | 33 (10.0%) | 78 (17.7%) | 23 (22.5%) |
| Gender, n (%) |  |  |  |  |  |  |
| Female | 392 (64.4%) | 88 (57.9%) | 129 (57.6%) | 181 (55.0%) | 207 (47.0%) | 50 (49.0%) |
| Male | 217 (35.6%) | 64 (42.1%) | 95 (42.4%) | 148 (45.0%) | 233 (53.0%) | 52 (51.0%) |
| Preoperative CLD, n (%) |  |  |  |  |  |  |
| No | 539 (88.5%) | 119 (78.3%) | 180 (80.4%) | 259 (78.7%) | 350 (79.5%) | 74 (72.5%) |
| Yes | 70 (11.5%) | 33 (21.7%) | 44 (19.6%) | 70 (21.3%) | 90 (20.5%) | 28 (27.5%) |
| Preoperative CVD, n (%) |  |  |  |  |  |  |
| NO | 521 (85.6%) | 128 (84.2%) | 183 (81.7%) | 282 (85.7%) | 320 (72.7%) | 58 (56.9%) |
| Yes | 88 (14.4%) | 24 (15.8%) | 41 (18.3%) | 47 (14.3%) | 120 (27.3%) | 44 (43.1%) |
| Preoperative DM, n (%) |  |  |  |  |  |  |
| No | 523 (85.9%) | 128 (84.2%) | 177 (79.0%) | 242 (73.6%) | 340 (77.3%) | 79 (77.5%) |
| Yes | 86 (14.1%) | 24 (15.8%) | 47 (21.0%) | 87 (26.4%) | 100 (22.7%) | 23 (22.5%) |
| Smoking history, n (%) |  |  |  |  |  |  |
| No | 437 (71.8%) | 105 (69.1%) | 165 (73.7%) | 245 (74.5%) | 366 (83.2%) | 81 (79.4%) |
| Yes | 172 (28.2%) | 47 (30.9%) | 59 (26.3%) | 84 (25.5%) | 74 (16.8%) | 21 (20.6%) |
| Drinking history, n (%) |  |  |  |  |  |  |
| No | 533 (87.5%) | 127 (83.6%) | 194 (86.6%) | 285 (86.6%) | 357 (81.1%) | 77 (75.5%) |
| Yes | 76 (12.5%) | 25 (16.4%) | 30 (13.4%) | 44 (13.4%) | 83 (18.9%) | 25 (24.5%) |
| Preoperative chemoradiotherapy, n (%) |  |  |  |  |  |  |
| No | 526 (86.4%) | 121 (79.6%) | 174 (77.7%) | 279 (84.8%) | 425 (96.6%) | 101 (99.0%) |
| Yes | 83 (13.6%) | 31 (20.4%) | 50 (22.3%) | 50 (15.2%) | 15 (3.4%) | 1 (1.0%) |
| ASA classification, n (%) |  |  |  |  |  |  |
| I | 0 | 0 | 1 (0.4%) | 0 | 0 | 1 (1.0%) |
| II | 513 (84.2%) | 121 (79.6%) | 169 (75.4%) | 263 (79.9%) | 423 (96.1%) | 99 (97.0%) |
| III | 89 (14.6%) | 27 (17.8%) | 45 (20.1%) | 58 (17.6%) | 17 (3.9%) | 2 (2.0%) |
| IV | 7 (1.2%) | 4 (2.6%) | 9 (4.1%) | 8 (2.5%) | 0 | 0 |
| Endoscopic surgery, n (%) |  |  |  |  |  |  |
| No | 509 (83.6%) | 136 (89.5%) | 183 (81.7%) | 237 (72.0%) | 439 (99.8%) | 86 (84.3%) |
| Yes | 100 (16.4%) | 16 (10.5%) | 41 (18.3%) | 92 (28.0%) | 1 (0.2%) | 16 (15.7%) |
| Skull base tumor, n (%) |  |  |  |  |  |  |
| No | 454 (74.5%) | 119 (78.3%) | 162 (72.3%) | 216 (65.7%) | 428 (97.3%) | 77 (75.5%) |
| Yes | 155 (25.5%) | 33 (21.7%) | 62 (27.7%) | 113 (34.3%) | 12 (2.7%) | 25 (24.5%) |
| Intracranial-extracranial communicating tumor, n (%) |  |  |  |  |  |  |
| No | 558 (91.6%) | 146 (96.1%) | 208 (92.9%) | 327 (99.4%) | 439 (99.8%) | 100 (98.0%) |
| Yes | 51 (8.4%) | 6 (3.9%) | 16 (7.1%) | 2 (0.6%) | 1 (0.2%) | 2 (2.0%) |
| Craniotomy, n (%) |  |  |  |  |  |  |
| No | 104 (17.1%) | 20 (13.2%) | 56 (25.0%) | 143 (43.5%) | 6 (1.4%) | 24 (23.5%) |
| Yes | 505 (82.9%) | 132 (86.8%) | 168 (75.0%) | 186 (56.5%) | 434 (98.6%) | 78 (76.5%) |
| Cerebral convexity tumor, n (%) |  |  |  |  |  |  |
| No | 439 (72.1%) | 88 (57.9%) | 135 (60.3%) | 214 (65.0%) | 204 (46.4%) | 82 (80.4%) |
| Yes | 170 (27.9%) | 64 (42.1%) | 89 (39.7%) | 115 (35.0%) | 236 (53.6%) | 19 (19.6%) |
| Supratentorial tumor, n (%) |  |  |  |  |  |  |
| No | 342 (56.2%) | 73 (48.0%) | 115 (51.3%) | 204 (62.0%) | 30 (6.8%) | 16 (15.7%) |
| Yes | 267 (43.8%) | 79 (52.0%) | 109 (48.7%) | 125 (38.0%) | 410 (93.2%) | 86 (84.3%) |
| Infratentorial tumor, n (%) |  |  |  |  |  |  |
| No | 508 (83.4%) | 125 (82.2%) | 195 (87.1%) | 289 (87.8%) | 406 (92.3%) | 90 (88.2%) |
| Yes | 101 (16.6%) | 27 (17.8%) | 29 (12.9%) | 40 (12.2%) | 34 (7.7%) | 12 (11.8%) |
| Pathology classification, n (%) |  |  |  |  |  |  |
| Brain metastases | 162 (26.6%) | 43 (28.3%) | 63 (28.1%) | 10 (3.0%) | 1 (0.2%) | 12 (11.8%) |
| Primary malignancy | 158 (25.9%) | 34 (22.4%) | 60 (26.8%) | 254 (77.2%) | 426 (96.8%) | 32 (31.4%) |
| Primary benign | 289 (47.5%) | 75 (49.3%) | 101 (45.1%) | 65 (19.8%) | 13 (3.0%) | 58 (56.8%) |
| Postoperative EVD, n (%) |  |  |  |  |  |  |
| No | 599 (98.4%) | 150 (98.7%) | 221 (98.7%) | 325 (98.8%) | 430 (97.7%) | 101 (99.0%) |
| Yes | 10 (1.6%) | 2 (1.3%) | 3 (1.3%) | 4 (1.2%) | 10 (2.3%) | 1 (1.0%) |
| SCD placement, n (%) |  |  |  |  |  |  |
| No | 156 (25.9%) | 28 (18.4%) | 58 (25.9%) | 81 (24.6%) | 1 (0.2%) | 23 (22.5%) |
| Yes | 453 (74.1%) | 124 (81.6%) | 166 (74.1%) | 248 (75.4%) | 439 (99.8%) | 79 (77.5%) |
| Reoperation, n (%) |  |  |  |  |  |  |
| No | 561 (92.1%) | 143 (94.1%) | 201 (89.7%) | 309 (93.9%) | 435 (98.9%) | 96 (94.1%) |
| Yes | 48 (7.9%) | 9 (5.9%) | 23 (10.3%) | 20 (6.1%) | 5 (1.1%) | 6 (5.9%) |

BMI, body mass index; GCS, glasgow coma scale; KPS, karnofsky performance status; Alb, serum albumin; Hgb, hemoglobin; Glu, serum glucose; CRP, C-reactive protein; MV, mechanical ventilation; EVD, extraventricular drainage; SCD, subcutaneous drainage; POP, postoperative pneumonia, CLD, chronic lung disease; CVD, cardiovascular disease; DM, diabetes mellitus; SIRI, systemic inflammatory response index; ASA, American Society of Anesthesiologists.

**Supplementary Table S3. Hyperparameters of ten candidate ML models.**

| Model | Implementation | Key hyperparameters |
| --- | --- | --- |
| Logistic Regression | sklearn.linear_model.LogisticRegression | penalty='none'; max_iter=100 |
| Naive Bayes | sklearn.naive_bayes.GaussianNB | priors=None; var_smoothing=1e-09 |
| Support Vector Machine | sklearn.svm.SVC | kernel='linear'; probability=True; max_iter=100 |
| Random Forest | sklearn.ensemble.RandomForestClassifier | n_estimators=5; max_depth=3; min_samples_split=4; random_state=0 |
| Extra Trees | sklearn.ensemble.ExtraTreesClassifier | n_estimators=60; max_depth=5; min_samples_split=2; random_state=0 |
| Gradient Boosting Machine | sklearn.ensemble.GradientBoostingClassifier | n_estimators=50; random_state=0 |
| AdaBoost | sklearn.ensemble.AdaBoostClassifier | n_estimators=10; random_state=0 |
| XGBoost | xgboost.XGBClassifier | n_estimators=6; objective='binary:logistic'; max_depth=3; min_child_weight=0.2; eval_metric='error'; use_label_encoder=False; random_state=0 |
| LightGBM | lightgbm.LGBMClassifier | n_estimators=2; max_depth=1; min_child_weight=0.5; random_state=0 |
| Multilayer Perceptron | sklearn.neural_network.MLPClassifier | hidden_layer_sizes=(61,128,64,32); solver='sgd'; max_iter=300; random_state=0 |
| Unless otherwise specified, all remaining hyperparameters were kept as the corresponding library defaults. | | |

**Supplementary Table S4. Performance of the ML models for POP prediction in the training and internal validation datasets.**

| Model | Accuracy | AUC (95% CI) | Sensitivity (95% CI) | Specificity (95% CI) | PPV (95% CI) | NPV (95% CI) | F1 (95% CI) | Brier score | Task |
| --- | --- | --- | --- | --- | --- | --- | --- | --- | --- |
| LR | 0.890 | 0.934 (0.904-0.964) | 0.860 (0.762-0.958) | 0.893 (0.866-0.920) | 0.454 (0.345-0.563) | 0.984 (0.972-0.992) | 0.594 (0.498-0.690) | 0.049 | Training |
|  | 0.862 | 0.942 (0.873-1.000) | 0.929 (0.858-1.000) | 0.855 (0.799-0.911) | 0.394 (0.209-0.571) | 0.992 (0.984-1.000) | 0.553 (0.345-0.703) | 0.036 | Internal validation |
| NB | 0.842 | 0.924 (0.891-0.957) | 0.877 (0.790-0.945) | 0.839 (0.810-0.869) | 0.360 (0.277-0.442) | 0.985 (0.975-0.994) | 0.510 (0.417-0.603) | 0.072 | Training |
|  | 0.875 | 0.930 (0.858-1.000) | 1.000 (1.000-1.000) | 0.862 (0.778-0.995) | 0.424 (0.260-0.591) | 1.000 (1.000-1.000) | 0.596 (0.393-0.752) | 0.063 | Internal validation |
| SVM | 0.737 | 0.847 (0.782-0.911) | 0.807 (0.757-0.857) | 0.730 (0.703-0.757) | 0.236 (0.190-0.282) | 0.973 (0.966-0.980) | 0.365 (0.308-0.422) | 0.073 | Training |
|  | 0.783 | 0.806 (0.657-0.955) | 1.000 (1.000-1.000) | 0.761 (0.648-0.874) | 0.298 (0.094-0.502) | 1.000 (1.000-1.000) | 0.459 (0.301-0.617) | 0.070 | Internal validation |
| RF | 0.895 | 0.950 (0.929-0.971) | 0.877 (0.810-0.944) | 0.897 (0.875-0.919) | 0.467 (0.381-0.553) | 0.986 (0.978-0.994) | 0.626 (0.529-0.711) | 0.051 | Training |
|  | 0.921 | 0.900 (0.789-1.000) | 1.000 (1.000-1.000) | 0.913 (0.830-0.996) | 0.538 (0.359-0.720) | 1.000 (1.000-1.000) | 0.605 (0.414-0.762) | 0.041 | Internal validation |
| ET | 0.947 | 0.979 (0.963-0.995) | 0.912 (0.813-0.990) | 0.951 (0.923-0.979) | 0.658 (0.548-0.764) | 0.991 (0.979-1.000) | 0.719 (0.623-0.800) | 0.039 | Training |
|  | 0.836 | 0.939 (0.871-1.000) | 1.000 (1.000-1.000) | 0.819 (0.743-0.895) | 0.359 (0.200-0.518) | 1.000 (1.000-1.000) | 0.528 (0.333-0.723) | 0.030 | Internal validation |
| XGBoost | 0.957 | 0.955 (0.918-0.993) | 0.930 (0.862-0.998) | 0.960 (0.943-0.975) | 0.707 (0.596-0.799) | 0.993 (0.979-0.996) | 0.803 (0.703-0.854) | 0.041 | Training |
|  | 0.921 | 0.921 (0.838-1.000) | 0.929 (0.750-1.000) | 0.920 (0.866-0.975) | 0.542 (0.367-0.725) | 0.992 (0.974-1.000) | 0.684 (0.484-0.839) | 0.037 | Internal validation |
| LightGBM | 0.719 | 0.838 (0.781-0.895) | 0.877 (0.783-0.958) | 0.703 (0.664-0.711) | 0.234 (0.177-0.294) | 0.982 (0.969-0.995) | 0.369 (0.294-0.443) | 0.080 | Training |
|  | 0.730 | 0.854 (0.753-0.954) | 0.857 (0.643-1.000) | 0.717 (0.553-0.714) | 0.235 (0.082-0.294) | 0.980 (0.949-1.000) | 0.369 (0.219-0.505) | 0.078 | Internal validation |
| GBM | 0.993 | 0.996 (0.990-1.000 | 0.965 (0.946-0.984) | 0.996 (0.988-0.999) | 0.965 (0.946-0.984) | 0.996 (0.988-0.999) | 0.965 (0.955-0.975) | 0.019 | Training |
|  | 0.868 | 0.957 (0.910-1.000) | 1.000 (1.000-1.000) | 0.855 (0.794-0.921) | 0.412 (0.267-0.575) | 1.000 (1.000-1.000) | 0.583 (0.404-0.742) | 0.012 | Internal validation |
| AdaBoost | 0.924 | 0.970 (0.956-0.984) | 0.895 (0.809-0.975) | 0.928 (0.902-0.950) | 0.560 (0.456-0.661) | 0.988 (0.977-0.996) | 0.689 (0.559-0.734) | 0.147 | Training |
|  | 0.842 | 0.944 (0.872-1.000) | 1.000 (1.000-1.000) | 0.826 (0.749-0.898) | 0.368 (0.239-0.504) | 1.000 (1.000-1.000) | 0.538 (0.372-0.686) | 0.149 | Internal validation |
| MLP | 0.921 | 0.944 (0.918-0.971) | 0.860 (0.756-0.944) | 0.928 (0.906-0.951) | 0.551 (0.412-0.633) | 0.985 (0.969-0.990) | 0.671 (0.538-0.733) | 0.047 | Training |
|  | 0.829 | 0.917 (0.833-1.000) | 1.000 (1.000-1.000) | 0.812 (0.748-0.876) | 0.350 (0.199-0.501) | 1.000 (1.000-1.000) | 0.519 (0.317-0.658) | 0.031 | Internal validation |

AUC, area under curve; PPV, positive predictive value; NPV, negative predictive value; LR, logistic regression, NB, NaiveBayes; SVM, support vector machine; RF, random forest; ET, ExtraTrees, XGBoost, extreme gradient boosting; LightGBM, light gradient boosting machine; AdaBoost, adaptive boosting; GBM, gradient boosting machine; MLP, multilayer perceptron.

**Supplementary Table S5. Performance of the top 5 ML models for POP prediction in the prospective test P dataset.**

| Model | Accuracy | AUC (95% CI) | Sensitivity (95% CI) | Specificity (95% CI) | PPV (95% CI) | NPV (95% CI) | F1 (95% CI) | Brier score |
| --- | --- | --- | --- | --- | --- | --- | --- | --- |
| GBM | 0.777 | 0.882 (0.818-0.944) | 0.865 (0.755-0.969) | 0.759 (0.694-0.821) | 0.416 (0.303-0.525) | 0.966 (0.936-0.993) | 0.561 (0.444-0.661) | 0.087 |
| ET | 0.848 | 0.866 (0.796-0.934) | 0.784 (0.639-0.903) | 0.861 (0.808-0.910) | 0.527 (0.400-0.654) | 0.953 (0.919-0.981) | 0.630 (0.506-0.727) | 0.095 |
| LR | 0.853 | 0.897 (0.842-0.952) | 0.784 (0.632-0.913) | 0.866 (0.813-0.912) | 0.537 (0.400-0.660) | 0.953 (0.920-0.983) | 0.637 (0.508-0.738) | 0.082 |
| AdaBoost | 0.893 | 0.896 (0.837-0.955) | 0.703 (0.543-0.842) | 0.931 (0.892-0.965) | 0.667 (0.514-0.814) | 0.941 (0.906-0.972) | 0.684 (0.548-0.800) | 0.169 |
| NB | 0.853 | 0.883 (0.819-0.947) | 0.811 (0.676-0.933) | 0.861 (0.808-0.909) | 0.536 (0.400-0.667) | 0.958 (0.926-0.987) | 0.645 (0.526-0.750) | 0.094 |

AUC, area under curve; PPV, positive predictive value; NPV, negative predictive value; GBM, gradient boosting machine; ET, ExtraTrees, LR, logistic regression; AdaBoost, adaptive boosting; NB, NaiveBayes.

**Supplementary Fig. S1 Heatmap of Spearman’s correlation analyses among variables.**

Features from the retrospective cohort were included in Spearman's correlation analysis, and the correlation coefficient values were displayed as a heat map.

BMI, body mass index; GCS, glasgow coma scale; KPS, karnofsky performance status; Alb, serum albumin; Hgb, hemoglobin; Glu, serum glucose; CRP, C-reactive protein; MV, mechanical ventilation; EVD, extraventricular drainage; SCD, subcutaneous drainage; POP, postoperative pneumonia, CLD, chronic lung disease; CVD, cardiovascular disease; DM, diabetes mellitus; ASA, American Society of Anesthesiologists; Systemic inflammatory response index, SIRI.

**Supplementary Fig. S2 Feature selection using the LASSO regression model.**

(A) LASSO coefficient profiles of candidate features plotted against the log(λ) sequence. The dashed line represents the optimal λ value selected via cross-validation, at which the model retains the most informative features and shrinking others to zero.

(B) Cross-validation curve for the LASSO model, showing the binomial deviance (mean ± standard error) as a function of log(λ). The dashed lines indicate the λ value minimizing the deviance (left) and the more conservative λ value within one standard error of the minimum (right).

(C) Coefficients of the selected features in the optimal LASSO model. Features include CLD, DM, preoperative chemoradiotherapy, BMI, smoking, preoperative Glu, postoperative Glu, ASA score, convexity tumor, preoperative Alb, and admission KPS.

CLD, chronic lung disease; DM, diabetes mellitus; BMI, body mass index; ASA, American Society of Anesthesiologists; KPS, karnofsky performance status.

**Supplementary Fig. S3 Distribution of predicted probability scores for top 5 ML models in distinguishing POP from non-POP samples.**

(A-E) Training set; (F-J) Internal validation set; (A, F) Gradient Boosting model; (B, G) Extra Trees model; (C, H) Logistic Regressio model; (D, I) AdaBoost model; (E, J) NaiveBayes model.

Each panel illustrates the sorted predicted probability scores for non-POP (blue) and POP (orange) samples. The x-axis represents the sample index (sorted by increasing predicted probability), and the y-axis represents the predicted probability of POP (ranging from 0 to 1). A clear separation between the blue and orange regions indicates better discriminative performance of the model.

**Supplementary Fig. S4 Precision-recall curves of top 5 ML models in distinguishing POP from non-POP samples in the prospective validation dataset.**

**Supplementary Fig. S5 Distribution of predicted probability scores for top 5 ML models in distinguishing POP from non-POP samples in the prospective validation dataset.**

Each panel illustrates the sorted predicted probability scores for non-POP (blue) and POP (orange) samples. The x-axis represents the sample index (sorted by increasing predicted probability), and the y-axis represents the predicted probability of POP (ranging from 0 to 1). A clear separation between the blue and orange regions indicates better discriminative performance of the model.

**Supplementary Fig. S6 SHAP dependence plot for the 11 features.** Each dot represents a sample and each dependence plot shows how a single feature affects the output of the prediction model.

SHAP: SHapley Additive exPlanation; KPS, karnofsky performance status; BMI, body mass index; CLD, chronic lung disease; DM, diabetes mellitus; ASA, American Society of Anesthesiologists.
